# Identifying Substance Use and High-Risk Sexual Behavior Among Sexual and Gender Minority Youth by Using Mobile Phone Data: Development and Validation Study

**DOI:** 10.2196/68013

**Published:** 2025-08-12

**Authors:** Mehrab Beikzadeh, Ian W Holloway, Kimmo Kärkkäinen, Chenglin Hong, Cory Cascalheira, Elizabeth S C Wu, Callisto Boka, Alexandra C Avendaño, Elizabeth A Yonko, Majid Sarrafzadeh

**Affiliations:** 1 Department of Computer Science, UCLA Samueli School Of Engineering, University of California, Los Angeles, 7400 Boelter Hall, Los Angeles, CA, 90034, United States, 1 4245664464; 2 Department of Social Welfare, University of California, Los Angeles, Los Angeles, CA, United States; 3 Optum, Los Angeles, CA, United States; 4 School of Social Work, University of Connecticut, Hartford, CT, United States; 5 Addiction Treatment Center, VA Puget Sound Health Care System, Seattle, WA, United States; 6 Department of Epidemiology, UCLA Fielding School of Public Health, University of California, Los Angeles, Los Angeles, CA, United States

**Keywords:** substance use, HIV risk, sexual and gender minoritized, mobile app, eHealth

## Abstract

**Background:**

Sexual and gender minority (SGM) individuals are at heightened risk for substance use and sexually transmitted infections than their non-SGM peers. Collecting mobile phone usage data passively may open new opportunities for personalizing interventions, as behavioral risks could be identified without user input.

**Objective:**

This study aimed to determine (1) whether passively sensed mobile phone data can be used to identify substance use and sexual risk behaviors for sexually transmitted infection (STI) and HIV transmission among young SGM who have sex with men, (2) which outcomes can be predicted with a high level of accuracy, and (3) which passive data sources are most predictive of these outcomes.

**Methods:**

We developed a mobile phone app to collect participants’ messaging, location, and app use data and trained a machine learning model to predict risk behaviors for STI and HIV transmission. We used Scikit-learn to train logistic regression and gradient boosting classification models with simple linear model specification to predict participants' substance use and sexual behaviors (ie, condomless anal sex, number of sexual partners, and methamphetamine use), which were validated using self-report questionnaires. *F*_1_-scores were used to quantify prediction accuracy of the model using different data sources (and combinations of these sources) for prediction. Differences between text, location, app use, and Linguistic Inquiry and Word Count (LIWC) domains by outcome were investigated using independent *t* tests where associations were considered significant at *P*<.05.

**Results:**

Among participants (n=82) who identified as SGM, were sexually active, and reported recent substance use, our model was highly predictive of methamphetamine use and having ≥6 sexual partners (*F*_1_-scores as high as 0.83 and 0.69, respectively). The model was less predictive of condomless anal sex (highest *F*_1_-score 0.38). Overall, text-based features were found to be most predictive, but app use and location data improved predictive accuracy, particularly for detecting ≥6 sexual partners. Methamphetamine use was significantly associated with dating app use (*P*=.01) and use of sex-related words (*P*=.002). Having ≥6 sex partners was associated with dating app use (0.02), use of sex-related words (*P*=.001), and traveling a further distance from home (*P*=.03), on average, compared to participants with fewer sex partners. Methamphetamine users were more likely to use social (*P*=.002) and affect words (*P*=.003) and less likely to use drive-related words (*P*=.02). People having 6 or more partners were more likely to use social, affect words, and cognitive process-related words (*P*=.003 and .004 respectively).

**Conclusions:**

Our results show that passively collected mobile phone data may be useful in detecting sexual risk behaviors. Expanding data collection may improve the results further, as certain behaviors, such as injection drug use, were quite rare in the study sample. These models may be used to personalize STI and HIV prevention as well as substance use harm reduction interventions.

## Introduction

Sexual and gender minoritized (SGM) individuals are at heightened risk for substance use and sexually transmitted infections (STIs) than the general United States population. Among SGM populations, men who have sex with men (MSM), for example, are twice as likely to use illicit drugs [1], which may be used to cope with negative life events and thoughts, or to enhance pleasure during sex [2]. Over half of new HIV infections occur among SGM, which can be attributed to sexual risk behaviors and intravenous drug use (IDU) [3-5]. Between 2018 and 2022, the Centers for Disease Control and Prevention reported HIV diagnoses increased significantly among transgender and gender nonbinary populations, more so than among cisgender men or women [5]. Research suggests that these health disparities in substance use and HIV are generated by unjust social conditions [6,7] and increased exposure to minority stressors [8,9]. SGM are also at higher odds of mental distress and depression [10,11], which in turn may increase substance use as a coping mechanism [2,12].

Systematic reviews of studies in SGM populations, largely thus far tailored for MSM, have shown that interventions can be effective on methamphetamine- and sexual health-related outcomes, such as condomless anal sex or substance use during sex [13], and participants find these interventions useful for gaining new knowledge and skills [14]. In addition, participants find interventions useful for self-reflection [14], which may lead to behavior change. However, results from a global survey among MSM who use substances found that only 11% of respondents had access to substance use treatment programs and only 5% participated in such a program [15]. In the United States, only 6.5% of people who needed substance use treatment received it in 2020 [16]. The majority of those who want substance use treatment but do not receive it experience significant access barriers such as affordability due to the lack of health care coverage, not finding an appropriate program, fear of others having a negative opinion of them, and the absence of culturally informed treatment tailored to the unique needs of SGM [17]. Therefore, efforts to address these disparities should prioritize improving access to health services for SGM, particularly strategies to deliver health services and identify those at the highest risk.

Mobile- and eHealth-based interventions could improve the accessibility of interventions, as they have potential to overcome many of these treatment barriers (eg, overcoming the stigma of receiving substance use treatment by using the eHealth intervention from the privacy of one’s home). Mobile and eHealth interventions may also open new opportunities for personalization through increased availability of data about participants. Prior studies have shown success in providing personalized HIV interventions to MSM and people using substances [18-20]. However, this personalization typically depends on participants reporting behaviors manually, which increases participant burden. For example, one study asked participants to respond to either daily or biweekly surveys, which many participants reported to be too repetitive [21]. Although burdensome, the group receiving daily surveys found them to be more useful than the group receiving biweekly surveys. This indicates that behavioral health monitoring should not depend on receiving frequent input from the participant. Therefore, being able to automate some or all the behavior monitoring could reduce participant burden.

In this study, we investigate how machine learning techniques can help identify sexual risk behaviors among SGM from passively sensed mobile phone data. Prior studies have predicted HIV risk using, for example, Twitter [22], electronic health records [23,24], or smartphone survey data [25]. Similarly, substance use risk has been detected using survey data [26], cognitive test results [27], Instagram (Meta) profile data [28], and social media posts [29]. To the best of our knowledge, this is the first study to correlate substance use and sexual risk behaviors among SGM using passively collected mobile phone data, which allows for frequent data collection with minimal effort required from the participant.

We first developed a mobile sensing app that tracks participants’ daily actions, such as their location, messaging, and app use. We then trained machine learning models to detect substance use and sexual risk behaviors from these data and evaluated their performance in predicting different behaviors. Finally, we analyzed how different risky behaviors manifest in mobile phone data.

The main contributions of this study are (1) demonstrating how passively collected mobile phone data can be used for behavioral risk prediction and identifying limitations of this approach; (2) evaluating which types of data should be collected to identify substance use and sexual risk behaviors by training machine learning models using different subsets of the data, as well as analyzing differences between participants’ data; and (3) Determining how accurately different behaviors can be identified from mobile phone data.

## Methods

### Design and Eligibility

Data for this analysis were derived from a National Institutes of Health–funded randomized comparison trial – uTECH (ClinicalTrials.gov identifier: NCT04710901). To be eligible for the study, participants had to meet the following criteria: (1) be 18 to 29 years old, (2) be able to speak in English, (3) identify as a sexual or gender minority, (4) have had anal or oral sex with a man in the past 3 months, (5) have used substances (such as alcohol, marijuana, poppers [amyl nitrate], methamphetamines, heroin, cocaine, and ecstasy) in the past 3 months, (6) have had sex while using substances in the past 3 months, (7) being HIV negative or of unknown HIV status, (8) have used a dating app to meet sexual and substance use partners in the past 3 months, (9) own a smartphone, (10) reside in the United States, (11) be willing to participate in a 12-month study, and (12) be able to provide informed consent. Eligibility criteria, recruitment procedures, and overall study design are detailed comprehensively in the protocol paper [30].

### Screening

All participants completed an initial screener survey that was hosted on Qualtrics, a web-based survey platform [31]. The screener provided information about the study and included questions to determine eligibility. If an individual met eligibility criteria, the survey used branching logic to show additional screens to ask for contact information, including the phone number of their smartphone [30].

Research staff took precautions against fraudulent screeners by using survey metadata to identify noncellular phone numbers, virtual private network software, and high-risk IP addresses [30]. Screeners that were suspected to be illegitimate were removed before enrollment in the study. SGM who completed the screener, met the eligibility criteria, and passed fraud detection checks were contacted to schedule a consent and onboarding session over the Zoom conferencing platform (Zoom Video Communications) [32].

### Ethical Considerations

**Figure 1.**
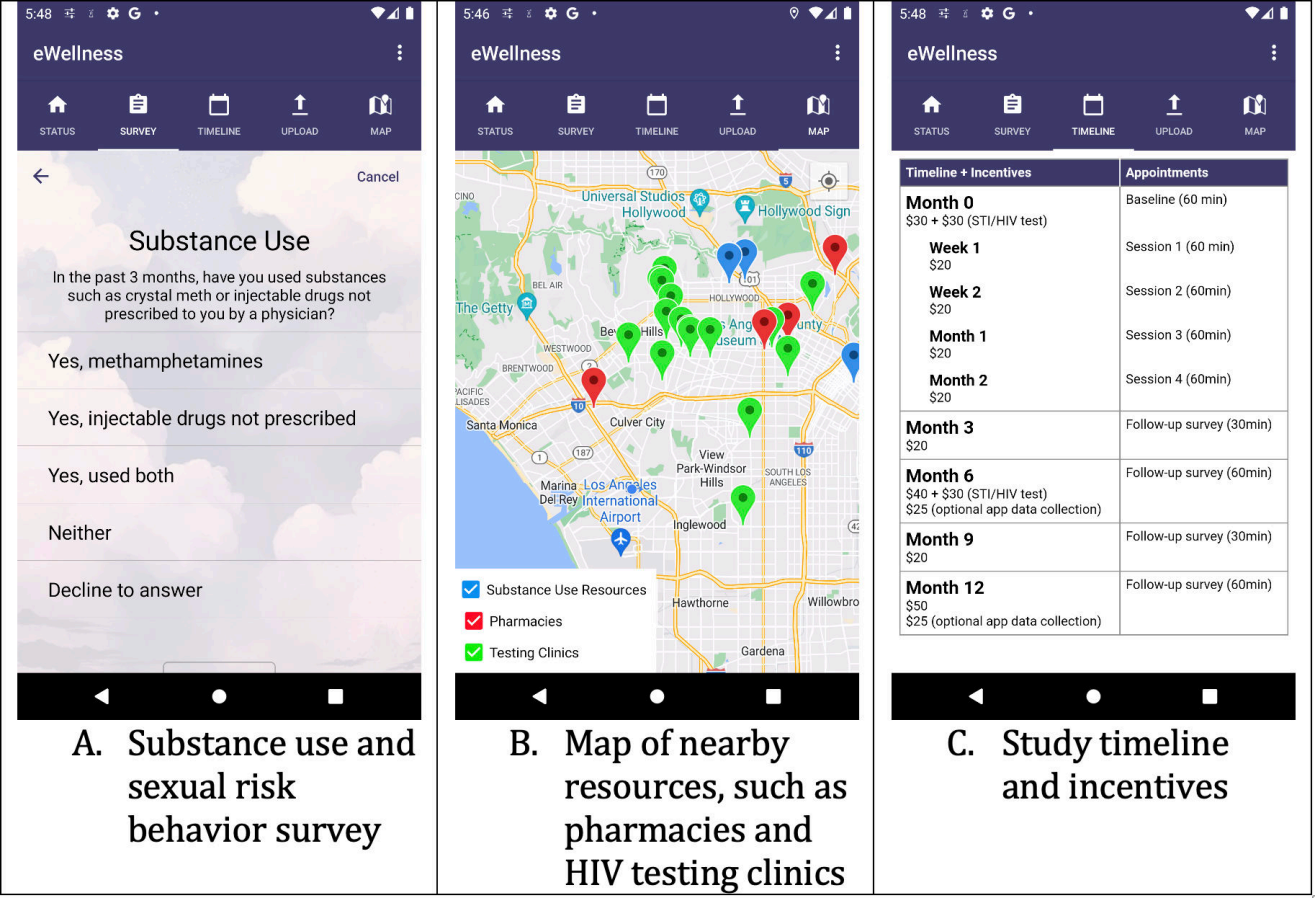
Screenshots of the eWellness app.

All study procedures and protocols were approved by the by the South Campus institutional review board of the University of California, Los Angeles (IRB#22‐000009). Informed consent was obtained during the onboarding process. During the informed consent process, the interviewer shared the consent document which provided details of what types of data (eg, keylogged data and Global Positioning System [GPS] data) were collected by the data collection app. The consent document also provided details on what kinds of data would not be collected (ie, photos and video). Benefits and risks of participation, monetary incentives, and data privacy protections were likewise detailed in the consent document. Participants were informed all data collected were protected from use as evidence in legal processes by a Certificate of Confidentiality granted by the National Institutes of Health (number: CC-OD-22‐3555). If the participant consented to participate, their agreement was recorded by the interviewer, and they were emailed a copy of the consent to keep for their records. Participants were compensated up to US $450 for completion of study-related activities (see Figure 1C for details).

All research staff were required to complete HIPAA (Health Insurance Portability and Accountability Act) and human subjects research training to gain access to participant materials. Any personally identifiable information or media collected unintentionally through data collection was redacted or removed entirely prior to storage in the study’s database.

### Data Collection App

Android [33] users who completed the consent and enrollment process would then install the data collection app used in the study, eWellness. The app was based on the Aware Framework [34], which has been used in numerous earlier eHealth studies, for example, to predict depression and anxiety [35,36], progression of Parkinson disease [37], or alcohol use events [38]. We adapted eWellness for Android phones to collect data on participants’ mobile phone use activities. We asked participants to give the app all the necessary permissions to passively collect keyboard and location data during participation. In addition to collecting keyboard data when participants typed text, the app collected information on which app they were typing the text in or, if they were using a browser, which website they were on.

The app also contained a substance use and sexual behavior survey, referred to as the “wellness survey,” (shown in Figure 1A) which the participants were asked to complete when they joined the study and once every 3 months after that. The survey was adapted from the US Centers for Disease Control and Prevention’s HIV and pre-exposure prophylaxis (PrEP) clinical practice guidelines [39]. The wellness survey contained questions on individual participants’ substance use and sexual risk behaviors, which yielded a total score indicating one’s risk for HIV infection and PrEP eligibility. The questions and answer options are shown in Table S1 in the Multimedia Appendix 1. To keep participants engaged, the app also provided other useful information, such as a map of nearby resources, such as pharmacies, HIV testing locations, and substance use harm reduction resources (Figure 1B), as well as the study timeline and incentives (Figure 1C).

The app sent the collected data to our secure server every 30 minutes whenever internet connection was available. Highly sensitive information, such as passwords, was filtered out and the research team had no access to them. The server-stored data did not contain other identifying information; only a randomly assigned participant identifier was included in the data. A document linking personal information necessary for participant follow-up was hosted on Federal Information Processing Standards 140‐2 certified cloud-based file management platform, Box. The Box directory was only accessible via multifactor authentication university-based single sign-on log-in to institutional review board–approved researchers with participant follow-up duties who had completed HIPAA and Good Clinical Practices trainings. All files containing participant information were protected with AES 256-bit encryption and data leak prevention and threat detection algorithms integrated into Box.

### Data Preprocessing

#### Keyboard Data

The eWellness app collected information about the currently active text field’s contents on every keystroke. As a result, our database contained multiple rows of data for every full line of text that the participant typed. For example, typing “Hello” might have been stored in the database as rows containing text values: “H,” “He,” “Hel,” “Hell,” and “Hello.” In addition, the participant could have changed earlier parts of the text or used autocorrection, which means that this same text could have appeared as database rows: “H,” “He,” “Hel,” “Helo,” “Hello.” As a result, the earlier row was not always a substring of the next one.

To remove duplicate rows, we repeated the following steps for each individual participant’s text data until there were no more rows to remove: (1) compare each row to the next row and if the first row is a substring of the second one, remove the first row, and (2) calculate the Levenshtein similarity between each row and the following row, and if the similarity is larger than 0.6, remove the first row.

Levenshtein similarity between two strings a and b is defined as:


(1) $$sim\left(a,b\right)=1-\;\frac{dist\left(a,b\right)}{max\left(len\left(a\right),\;len\left(b\right)\right)}$$

where dist(a,b) is the Levenshtein distance [40] which counts the minimum number of single-character modifications (insert, delete, substitute) that are necessary to make the strings identical:


(2) $$dist\left(a,b\right)=\;\left\{ \begin{array}{l} len\left(a\right),\;if\;len\left(b\right)=0,\;or\;vice\;versa\\ dist\left(tail\left(a\right),\;tail\left(b\right)\right),\;if\;a\left[0\right]=b\left[0\right]\\ 1+min\left(dist\left(tail\left(a\right),\;b\right),\;dist\left(a,\;tail\left(b\right)\right),\;dist\left(tail\left(a\right),\;tail\left(b\right)\right)\right) \end{array}\right.$$

where *a[0]* refers to the first character of string *a*, and *tail(a*) refers to a substring of *a* which contains everything except the first character. We calculated the similarity score using the TextDistance Python library [41].

#### Application Data

For every row of text data, we had a package name of the app where the text was entered as well as the Uniform Resource Locator (URL) of the website if the participant was using a web browser. In many cases, online services can be accessed both through an app and through a website, so we combined these data sources by extracting the domain name from the URL and mapping the commonly appearing URLs to the corresponding package names using a manually curated list of domain name or app pairs. If a domain name was not in this list, the domain name itself was used as the package name. Apps and websites were treated in the same manner in analysis and model training.

#### Location Data

The eWellness app saved GPS coordinates periodically whenever the phone moved to a different location. The app avoided unnecessary data collection to reduce battery consumption by only collecting location data when the phone was moving. This meant that if the participant remained in the same location, we did not receive location data until the participant started moving again. As we were only interested in locations in which the participant spent time rather than locations that the participant moved by, we removed data points where the participant was moving and only retained the last location once the movement ended.

### Feature Extraction

After cleaning the dataset using the previously shown steps, we manually extracted various features (ie, computed independent variables) from each of the data sources to be used with the machine learning algorithms. These feature extraction techniques are described in the following subsections.

#### Text Data

In our dataset, participants were active for different numbers of days, and for individual participants, different days had sometimes vastly different amounts of text data. This made the direct application of traditional text processing techniques challenging. In addition, the words and phrases used by participants sometimes differed from the ones used by the general public, so for optimal results, the techniques had to be tailored for the study population. We only considered text data collected from social media, dating, or messaging apps/websites, as text data from other sources was found to contain more noise than useful information (for example, product names in shopping apps or location names in navigation apps).

The first set of features extracted from the text data was the frequencies of individual words used. Participants’ text was first lemmatized, which means that inflected word forms were transformed to their base forms (eg, “walking” → “walk,” “better” → “good”) to avoid having the same word appear in multiple forms in our set of features. Lemmatization was performed using WordNet Lemmatizer from the Natural Language Toolkit (NLTK) [42]. Then, we removed words defined in NLTK’s stop word list, commonly appearing placeholder texts (eg, “Enter message,” “Say something”), as well as words used by fewer than five people. For each remaining word, we calculated the frequency of word use:


(3) $$freq\left(w\right)=\frac{\#\;days\;with\;word\;w}{\#\;days\;with\;text\;data}$$

The second set of text features only considered words and phrases associated with drug use or sexual behaviors. We used a phrase list, which had been found effective for identifying HIV risk behavior as well as substance use by an earlier study [29], and we used our previously defined frequency formula to determine frequencies for both individual phrases as well as for higher-level phrase categories (ie, different types of substance use or sexual behavior).

A third set of features was computed by Linguistic Inquiry and Word Count (LIWC) software [43], which uses built-in dictionaries to capture social and psychological states. It computes features describing how much an individual talks about a variety of topics, such as money, physical intimacy, or leisure activities, and it also computes higher-level descriptive features to measure factors, such as analytical thinking, authenticity, and emotional tone. It has been used in numerous studies to, for example, analyze fake news [44], social media posts [45,46], online reviews [47], and college admission essays [48]. We used it to generate features for each individual day, and we calculated the average across all days for each individual participant.

Our last set of text features was generated using the Bidirectional Encoder Representations from Transformers (BERT) language model [49]. We used a model that was pretrained for sentiment analysis using Twitter data [50], as we expected Twitter data to use similar language as other social media and messaging platforms. We removed the last fully connected layer of the model so that it could be used to generate text embeddings, and we applied it to each individual day of data. These text embeddings were then averaged across all days of data for individual participants.

#### App Data

We captured app usage by looking at apps where the user wrote text. We generated one set of features by calculating how frequently each app was used:


 (4) $$freq\left(app\right)=\frac{\#\;days\;using\;app}{\#\;days\;using\;any\;apps}$$

We also considered a subset of these frequency features that only contained social media, dating, and messaging apps, as we expected other types of apps to be less relevant to our prediction task. Other apps (eg, maps, music, or shopping) were expected to be noisy and therefore to have a negative effect on the model’s predictive performance.

#### Location Data

Before extracting features from location data, we clustered GPS coordinates for each individual participant by using the Mean-Shift algorithm [51]. This algorithm moves all points repeatedly towards the mean value of their neighborhood (determined by window radius r) until all points have converged. Points that converge to the same coordinates are defined to belong in the same cluster, thus allowing the algorithm to find the appropriate number of clusters. As the generated clusters depend on the window size, we determined the appropriate size by visually inspecting the clustering results. We also assumed that the most visited location was the participant’s home.

We then computed the features describing individuals’ mobility, such as how far from home they traveled, how many locations they visited per day, and how many of these locations were unique. These features were selected such that they were potentially related to participants' behavioral health outcomes either directly or indirectly. For example, several unique locations may be associated with having many partners, while having very few unique locations could be related to methamphetamine use due to the limited number of locations where the participant could safely use the drug. The full list of location-based features is shown in Multimedia Appendix 1.

### Model Training

We used Scikit-learn [52] to train logistic regression [53] and gradient boosting [54] classification models to predict participants’ answers to each survey question. These models were chosen to represent a simple linear model as well as a more advanced nonlinear model. To determine which types of data could be useful for the prediction task, we trained separate models using individual data categories, such as location data, app use, and risky word use. We then evaluated combinations of these features, focusing on feature combinations that we believed would give a comprehensive view of the participant’s activities without including redundant data (eg, not including social media apps and all apps in the same model). Models were evaluated using leave-one-out cross-validation due to the relatively small number of participants.

To address class imbalance, we calculated *F*_1_-scores for the minority class and used gradient boosting which can better handle imbalanced datasets. After initial exploration of model hyperparameters, we selected values that provided stable performance. For logistic regression, we used default hyperparameters with max_iter=1000 to ensure convergence. For gradient boosting, we used a GradientBoostingClassifier with n_estimators=80 and default values for other hyperparameters.

We chose to focus on *F*_1_-scores for model evaluation, as they balance precision and recall considerations. In the context of behavioral risk prediction for potential interventions, both types of misclassification errors have important implications: false positives might lead to unnecessary interventions, while false negatives could miss individuals who might benefit from support. The *F*_1_-score helps balance these considerations for our exploratory analysis, though future applications may need to adjust classification thresholds based on specific intervention contexts.

## Results

### Study Population

Sociodemographic, sexual risk, and substance use characteristics reported by participants (n=82) at baseline are summarized in Table 1.

**Table 1.** Self-reported participant characteristics at baseline.

Characteristic	Total
Participants, n (%)	82 (100)
Age, mean (SD)	25.2 (3.9)
Race and ethnicity, n (%)	
American Indian or Native Alaskan	2 (2.4)
Asian	13 (15.9)
Black or African American	7 (8.5)
Hispanic or Latino	10 (12.2)
Middle Eastern and North African	1 (1.2)
Two or more races	11 (13.4)
Non-Hispanic White	38 (46.3)
Gender identity, n (%)	
Cisgender man	54 (65.9)
Transgender man	14 (17.1)
Nonbinary	11 (13.4)
Transgender woman	3 (3.7)
Sexual orientation, n (%)	
Gay	55 (67.1)
Bisexual or Pansexual	20 (24.4)
Queer	5 (6.1)
Straight or heterosexual	1 (1.2)
Refuse to answer	1 (1.2)
Education, n (%)	
Less than college degree	33 (40.2)
College degree or higher	48 (58.5)
Refuse to answer	1 (1.2)
US region, n (%)	
West	26 (31.7)
Northeast	25 (30.5)
South	17 (20.7)
Midwest	14 (17.1)
Sexual behavior, n (%)	
Condomless receptive sex	61 (74.4)
Condomless insertive sex with HIV+ partner 5+ times	3/74 (4.1)
HIV+ partners	8/74 (10.8)
6+ partners	41 (50)
11+ partners	26 (32)
Substance use, n (%)	
Methamphetamine use	15 (18.3)
Injection drug use	3 (3.7)
Injects cocaine	1 (1.2)
Injects in group	2 (2.4)
Shares injection equipment	1 (1.2)
Injects methamphetamine	2 (2.4)
In substance use treatment program	0 (0)

### Data Statistics

We collected data from participants between November 10, 2021, and April 15, 2024. Dataset statistics are shown in Table 2. Among all the apps, we manually identified 68 social media, dating, and messaging apps which were later used to analyze participants’ messaging data. It should be noted that apps included unique websites as well (grouped by domain name).

We used data for all participants who had at least 30 days of data available. If a participant’s answer to a survey question was “Decline to answer” or “I don’t know,” this answer was not included in the model training or evaluation. This did not, however, exclude their other survey answers from being used. Statistics for survey responses are shown in Table 1 (full questions and answer options are shown in Table S2 in Multimedia Appendix 1).

**Table 2.** Text, location, and app use summary statistics.

	Total	Mean	Median	SD	Min	Max
Lines of text	4,848,639	59,129.7	45,758	43,832.2	2529	195,797
Locations	1,607,149	16,917.4	6071	26,793	509	169,334
Unique apps	2248	92.1	89.5	46.4	20	289

### Model Performance

We trained classification models to predict answers to each question. As some questions had partially overlapping answer options, we split them into multiple distinct questions. For example, the answer options for substance use included methamphetamine use, injection drug use, and both, so we split it into two questions: methamphetamine use and injection drug use. In addition, some questions had a very low number of positive responses (for example, only 2 participants were in a substance use treatment program). As a result, the focus of our discussion will be on the three questions which we determined to be the most informative: methamphetamine use, having 6 or more male sexual partners, and receptive anal sex without a condom.

Results for predicting survey responses using both individual feature types as well as combinations of them are shown in Table 3. Feature combinations were selected both based on their individual results and based on whether they were presumed to provide nonoverlapping information. For example, we avoided combining highly correlated feature groups, such as social media apps and all apps, in the same model.

As the results show, methamphetamine use could be predicted well using just the text data. The word frequency feature with the gradient boosting model worked best. Predicting having many partners worked reasonably well when combining all feature types. Predictive models were only moderately successful in determining whether the participant had receptive condomless anal sex.

Combining multiple feature types rarely improved the performance by a noticeable amount. This could be because in many cases, the feature groups might provide redundant information, so using only one highly informative feature group was enough. In addition, increasing the number of features could lead to overfitting, as the number of features can become much larger than the number of participants.

**Table 3.** *F*_1_-scores for predicting answers to survey questions. *F*_1_-score was calculated for the less frequent response, which in most cases was the “positive” answer (answer frequencies are shown in Table 1). The first value shows the score using logistic regression and the second value shows the score using a gradient-boosting classifier.

	Methamphetamine use	Sexual behavior	
		6+ partners	Condomless receptive sex
Social apps	0.32/0.40	0.49/0.53	0.35/0.16
All apps	0.31/0.08	0.56/0.60	0.29/0.33
Location	0.00/0.25	0.46/0.39	0.00/0.00
Risky words	0.48/0.52	0.62/0.65	0.29/0.12
All words	0.29/0.83^a^	0.57/0.23	0.22/0.11
LIWC^b^	0.52/0.47	0.61/0.63	0.31/0.26
Bert	0.34/0.34	0.67/0.64	0.30/0.38^a^
Risky words, LIWC	0.50/0.67	0.62/0.61	0.32/0.25
Social apps, Risky words	0.48/0.38	0.58/0.65	0.28/0.15
Social apps, Risky words, LIWC	0.48/0.67	0.60/0.55	0.33/0.31
Social apps, Risky words, Location	0.52/0.48	0.61/0.69^a^	0.24/0.17
Social apps, Risky words, Location, LIWC,	0.52/0.67	0.61/0.64	0.19/0.07
All	0.42/0.58	0.62/0.49	0.10/0.21

a Highest predictive result for the corresponding outcome.

b LIWC: Linguistic Inquiry and Word Count

### Feature Analysis

**Figure 2.**
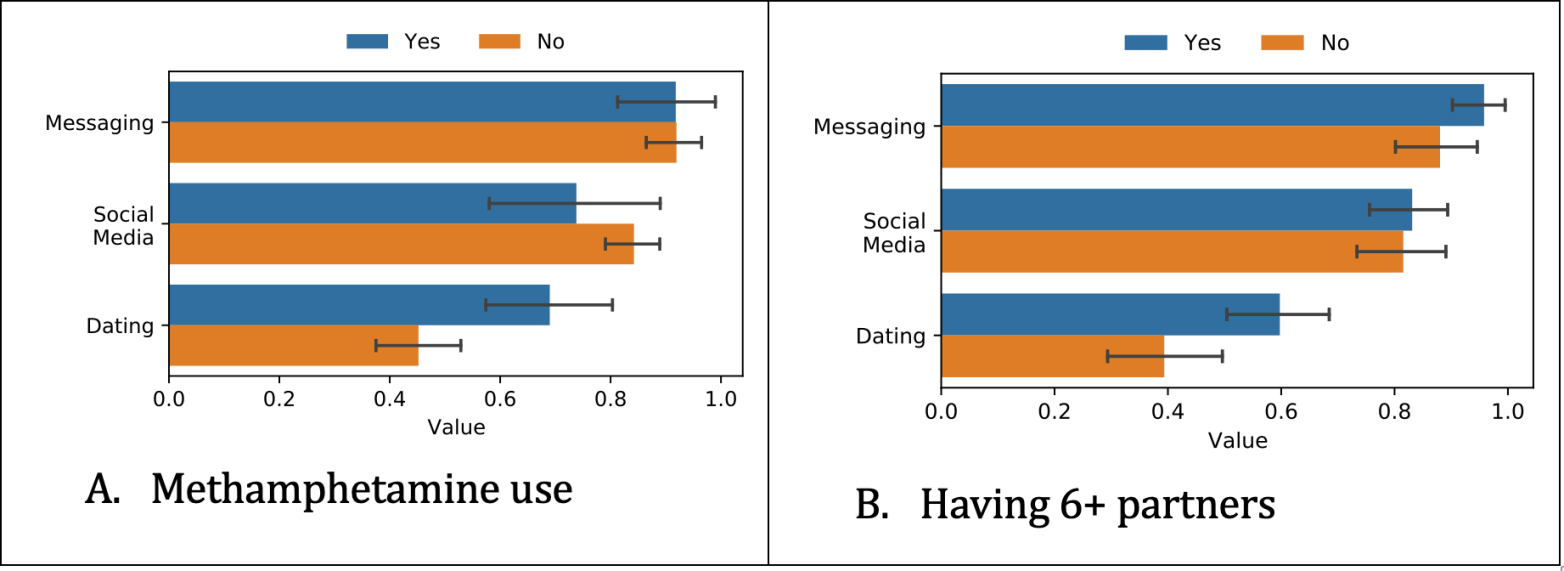
Differences in app use among different groups. The x-axis represents the percentage of days when the participant communicated using an app from a certain category. The blue bar corresponds to “Yes” methamphetamine use or “Yes” had ≥6 partners, respectively.

**Figure 3.**
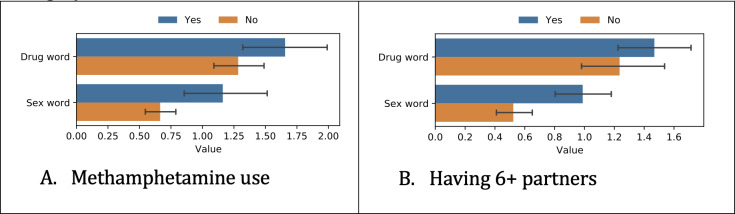
Differences in risky word use among different groups. The x-axis represents the percentage of days when the participant used words or phrases from a certain category. The blue bar corresponds to “Yes” methamphetamine use or “Yes” had ≥6 partners, respectively.

**Figure 4.**
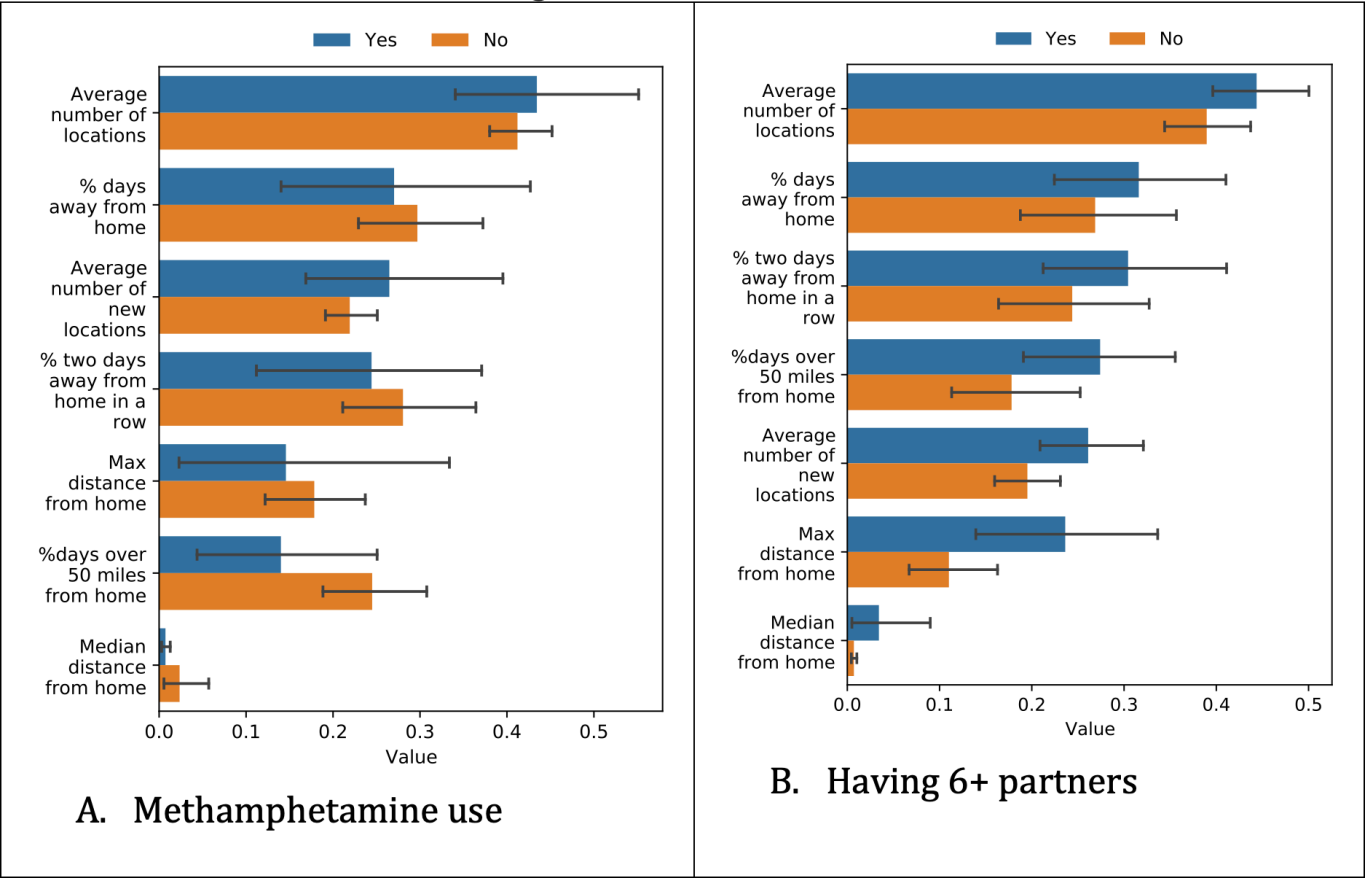
Differences in location data among different groups. Values have been scaled such that the largest individual value for each feature becomes 1 to be able to show all values in the same figure. The blue bar corresponds to “Yes” methamphetamine use or “Yes” had ≥6 partners, respectively.

Next, we analyzed how the participant data differed depending on the survey responses. We show the differences based on independent *t* tests for the most predictive tasks (Figures 2-4), which were methamphetamine use and having ≥6 partners.

#### App Use

Figure 2 shows how frequently participants used apps from different categories. We considered any apps that are used for communicating with other people and divided them into 3 categories: messaging apps (eg, Messages, WhatsApp, and Telegram), social media apps (eg, Facebook, Instagram, Twitter [currently known as X]), and dating apps (eg, Grindr, Tinder, and Adam4Adam).

Methamphetamine users or participants who had ≥6 partners were more likely to use dating apps than nonusers (*P*=.01, and *P*=.02, respectively). However, differences in the use of social media and messaging apps were not statistically significant for these groups.

#### Risky Words

Figure 3 shows differences in risky word use. We divided the list of risky words into sex-related and drug-related words.

Methamphetamine users and individuals with 6+ partners were both more likely to use sex-related words, with significance levels of *P*=.002 and *P*=.001, respectively. However, the difference in drug-related word usage was not statistically significant for methamphetamine users and individuals with 6+ partners. (*P*=.12 and .21, respectively)

#### Location

Figure 4 shows how location data differed for different groups. Due to the large number of location-based features, we chose a smaller subset of features that contained less overlapping information.

Methamphetamine users were less likely to spend time over 50 miles from home, although the difference was not statistically significant (*P*=.14). People with 6+ partners were found to travel further than those with 5 or fewer partners (*P*=.03).

#### LIWC

**Figure 5.**
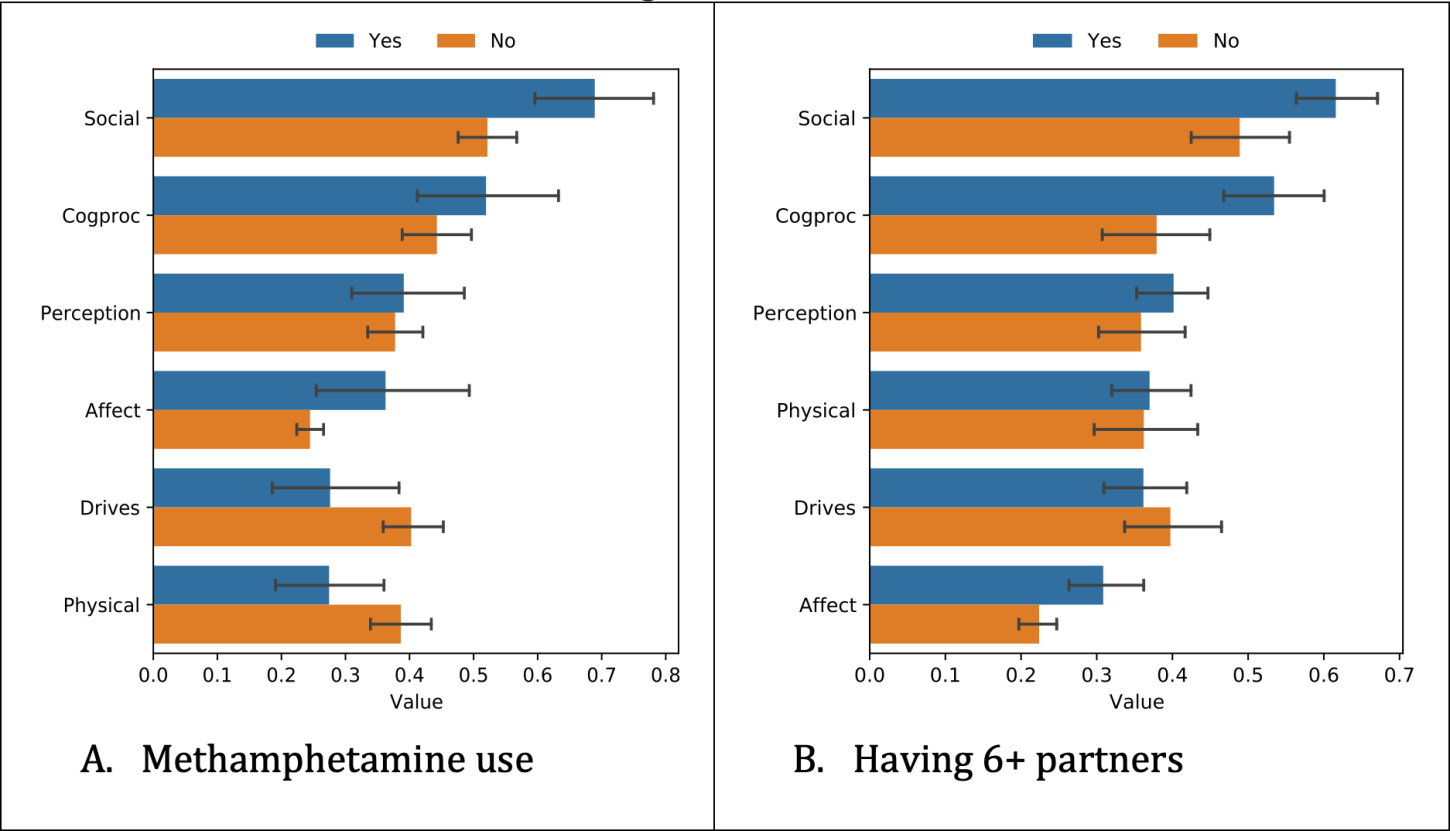
Differences in Linguistic Inquiry and Word Count features among different groups. Original values have been scaled to fit in the same figure.

Lastly, we compared LIWC features among different groups (Figure 5). Again, due to the large number of distinct features, we show results only for some of the super-categories which we expected to show differences.

Methamphetamine users were more likely to use social (*P*=.002) and affect words (*P*=.003) and less likely to use drive-related words (*P*=.02). People having 6 or more partners were more likely to use social, affect words, and cognitive process-related words (*P*=.003 and .004 respectively).

## Discussion

### Principal Results

In this paper, we have shown that mobile sensing data can be used to identify multiple risk behaviors of SGM in our study sample. More specifically, our participants’ text and location data were highly informative of methamphetamine use and having over 6 sexual partners in three months.

In addition to determining which behaviors can be predicted, our second goal was to determine what data is useful for these predictions. We have shown that text-based features like all words, risky words, and BERT were the most informative for most behaviors, which was an expected result because participants might, for example, look for partners on dating apps or discuss substance use in private messages with other people. This is aligned with previous research [29] showing that certain types of substance use may be predicted from social media messaging data.

In addition, we have shown that more recent language modeling techniques, such as BERT, can often provide similar results as the traditional techniques based on predetermined word lists and word frequencies. However, the more abstract nature of these representations may complicate the interpretation of the results, as individual values do not have a human-interpretable meaning. This lack of human interpretability might not be a limitation in digital health applications, where the emphasis is on achieving high precision and recall for effective intervention delivery rather than on understanding the underlying phenomena. On the other hand, BERT representations can also help improve privacy, as they do not reveal which exact words the participants used. Due to the small number of participants, training a language model using our dataset was not feasible, so we relied on a model that had been trained on Twitter (currently known as X) data. While we expect the language use to be mostly similar across datasets, training a language model using data from the target population could improve the results if enough data were available, as people might use different words and phrases on Twitter compared to dating apps or private messages.

We were also able to detect behavioral differences between groups of people with different survey responses. For example, methamphetamine users were more likely to use sex-related words in their messages. Earlier research has shown that methamphetamine users have more sexual partners [55] and may be engaged in more risky sexual behavior, such as condomless anal sex, although it is not clear whether the relationship between methamphetamine use and condomless anal sex is causal [56]. Methamphetamine users were also less likely to travel far from home. This may be related to lower household income level [57], which could make traveling far unaffordable, or paranoia induced by methamphetamine use. They also used more affective and social words and fewer drive-related words, which may be partly related to the higher prevalence of co-occurring mental health problems [57]. These insights could be valuable in personalizing methamphetamine use harm reduction and treatment by crafting prevention messages that frame behavioral modification as an affective or social process, instead of, for example, focusing on motivation.

We also found that participants with greater than 6 sex partners were more active users of all types of social apps (messaging, social media, and dating). Earlier studies have shown that users of geosocial networking apps, such as Grindr, have more sexual partners in general [58,59], and SGM with more partners have larger social networks [60], which may explain the more frequent use of social apps. Being more social may similarly explain more time spent away from home and in many different locations. People with greater than 6 sexual partners also used more sex- and drug-related words. Substance use has previously been found to be associated with a larger number of sexual partners [61]. These findings point toward personalized prevention that leverages both social media platforms and geolocation data. Partnerships between public health and dating apps to date have been limited to advertising and the addition of profile features (eg, fields for PrEP use). Researchers and public health practitioners might explore partnerships that allow users to opt into health promotion campaigns that are personalized by app use. Sexual health and substance use harm reduction should be pushed in relation to users’ geolocation.

### Comparison With Previous Work

The closest work to ours identified HIV as well as amphetamine, methamphetamine, and tetrahydrocannabinol use from social media messaging data [29]. Our work differs from this by using a wider range of data sources collected through participants’ mobile devices. For example, we used text typed in any mobile app and website which allowed us to identify sexual risk behaviors in traditional messaging apps and less common dating apps in addition to the most popular social media apps. In addition, we used location data, which allows us to analyze participants’ daily movement patterns and their relation to risk behaviors. We also attempted to identify a wider range of risk behaviors, especially related to sexual health. The only shared prediction target between these 2 studies was methamphetamine use; the earlier paper was able to predict with an *F*_1_-score of 0.85, which was very close to our result (0.83). In summary, our work is aligned and expands previous research on this topic.

Another similar study predicted alcohol, tobacco, prescription drug, and illegal drug use from Instagram data [28]. They were able to detect alcohol use with statistical significance, but they had less success in predicting other types of substance use. Our better prediction results may be attributed to having access to more personal messaging data, as many people may avoid discussing substance use on public platforms. This shows that choosing the appropriate data collection methods is very important for accurate results.

Other studies have implemented personalized MSM interventions using survey data [62], identified the efficacy of MSM-targeted mobile app interventions [63], or evaluated the feasibility and acceptability of mobile sensing among MSM [64-66]. However, these studies have not evaluated whether mobile sensing data can be used to inform and personalize interventions, nor have they incorporated a broader SGM population inclusive of transgender and gender-diverse individuals, which were the goals of our study.

### Limitations

A limitation of our study was that we only included participants who had an Android smartphone. We chose to only include Android users because iPhones have more restrictions on what data can be collected, and therefore collecting text data would have been unfeasible based on the budget for this project. The demographic differences between Android and iPhone users have been previously described, with iPhone users more likely to be female, younger, more concerned about their smartphone as a “status object,” and displaying lower levels of honesty and humility and higher levels of emotionality [67]. The restriction of our study population to Android users only may limit the generalizability of results from this formative study. Potential participants had to be excluded from this study either because of the challenges with iOS adaptation (n=324) or due to missing data from Android users (n=11), which may skew the demographics to some extent, again impacting generalizability. In addition, as the data was collected using personal devices, there were some interruptions in data collection. For example, some participants turned off or deleted the app during the study while others upgraded to a new phone without reinstalling the app. Some Android phones were found to have aggressive battery-saving functionality which occasionally turned off the data collection. To avoid data collection issues, we kept track of when each participant’s device had last sent us data and contacted participants after three missing days to make sure data collection could be resumed.

Our machine-learning model was trained on English-language text data only, which limits its ability to accurately interpret text written in other languages or in culturally specific dialects and vernaculars. While the ability to speak and understand English was one of our eligibility criteria, we did not exclude participants who are multilingual (ie, speak one or more languages other than English), and the model may not accurately process multilingual input. This limitation highlights the need for our future work to incorporate multilingual natural language processing approaches to better reflect diversity of language use.

Another limitation was that the text data only included what the participants typed on their phones. This approach may miss the context of some messages, as the responses are not collected. It could be informative to know what content participants consumed online or what messages they received from others. In addition, participants might have messaged with people using multiple devices (eg, computer or tablet in addition to their phone), so our data collection approach might not have been able to track all social media usage and messaging for some participants.

Finally, some of the outcomes that we set out to predict were very infrequent, which made the task impossible. For example, only two of our participants were in a substance use treatment program, which was not enough for training and evaluating a machine learning model. Therefore, we had to focus on questions that had a reasonable number of both positive and negative responses.

### Conclusions

In this study, we have shown that certain types of substance use and sexual risk behaviors can be determined from data that is collected from smartphones passively. Next, we demonstrated these data to be highly predictive of self-reported methamphetamine use and having 6 or more sexual partners. If integrated into downstream eHealth/mHealth interventions, passive mobile-sensing could be used to personalize interventions for SGM, which may reduce the burden of participating in intervention programs, as the daily behaviors can be tracked with minimal effort from the participant. However, further work is still needed to evaluate the efficacy of interventions based on automatic behavior tracking.

Our future work will explore providing personalized interventions using predictive models to determine which types of interventions may be appropriate. We will, for example, investigate sending participants messages and resources that are delivered “just in time,” such as providing information about PrEP to individuals who may be at elevated HIV risk based on their substance use or sexual behavior but who are not yet taking it. This work will include developing interpretation guidelines for both automated systems and health care providers who may use these predictions in clinical settings.

## Supplementary material

10.2196/68013 Multimedia Appendix 1 Risk assessment survey and location-based features.

## Abbreviations

BERT: Bidirectional Encoder Representations from Transformers
HIPAA: Health Insurance Portability and Accountability Act
IDU: Intravenous Drug Use
LIWC: Linguistic Inquiry and Word Count
MSM: men who have sex with men
NLTK: Natural Language Toolkit
PrEP: pre-exposure prophylaxis
SGM: sexual and gender minority
STI: sexually transmitted infection
