# Supplementary material for: Identifying Substance Use and High-Risk Sexual Behavior Among Sexual and Gender Minority Youth by Using Mobile Phone Data: Development and Validation Study
Source: Online J Public Health Inform. 2025 Aug 12;17:e68013. doi: 10.2196/68013 (PMC12360732; doi:10.2196/68013)
Supplement: Multimedia Appendix 1 [file ojphi-v17-e68013-s001.docx]

## Multimedia Appendix 1

### Risk Assessment Survey

Table S1. Sexual behavior and substance use survey

| Question | Answer options |
| --- | --- |
|  |  |
| How old are you today? | 1. (Enter number) |
| In the past 3 months, have you used substances such as crystal meth or injectable drugs not prescribed to you by a physician? | 1. Yes, methamphetamines 2. Yes, injectable drugs not prescribed 3. Yes, used both 4. Neither 5. Decline to answer |
| In the past 3 months, were you in a substance use in-patient or out-patient treatment program? | 1. Yes 2. No 3. Decline to answer |
| In the past 3 months, did you inject cocaine? | 1. Yes 2. No 3. Decline to answer |
| In the past 3 months, did you inject methamphetamines? | 1. Yes 2. No 3. Decline to answer |
| In the past 3 months, did you use needles? | 1. Yes 2. No 3. Decline to answer |
| In the past 3 months, did you share injection equipment? | 1. Yes 2. No 3. Decline to answer |
| In the past 3 months, did you inject in a group setting? | 1. Yes 2. No 3. Decline to answer |
| Do you currently take PrEP? PrEP stands for pre-exposure prophylaxis and it is a medication that helps prevent HIV transmission. | 1. Yes 2. No 3. Decline to answer |
| In the last 3 months, how many men have you had sex with? | 1. >10 2. 6-10 3. 1-5 4. 0 5. Decline to answer |
| In the last 3 months, how many times did you have receptive anal sex (you were the bottom) with a man when he did not use a condom? | 1. 1 or more times 2. 0 times 3. Decline to answer |
| In the last 3 months, how many of your male sex partners were HIV-positive? | 1. More than 1 HIV+ male partners 2. 1 HIV+ male partner 3. 0 4. Don’t know 5. Decline to answer |
| In the last 3 months, how many times did you have insertive anal sex (you were the top) with a man who was HIV-positive when you did not use a condom? | 1. 5 or more times 2. 0-4 times 3. Decline to answer |

### Location based features

Table S2. Features computed from the location data

| Metric |
| --- |
|  |
| 1. Maximum/mean/median distance from home |
| 1. Percentage of days spent over 10 or over 50 miles away from home |
| 1. Percentage of days (or 2 or 3 consecutive days) spent entirely away from home |
| 1. Percentage of days spent away from two or three of the most common locations |
| 1. Percentage of days with more than one or two location(s) |
| 1. Average number of locations visited |
| 1. Number of unique locations visited |
